# Temporomandibular Joint Disorders: Functional and Conservative Treatment

**DOI:** 10.3390/jcm12144772

**Published:** 2023-07-19

**Authors:** Luigi Angelo Vaira, Giacomo De Riu

**Affiliations:** 1 Maxillofacial Surgery Operative Unit, Department of Medicine, Surgery and Pharmacy, University of Sassari, 07100 Sassari, Italy; gderiu@uniss.it; 2 Biomedical Science Department, PhD School of Biomedical Science, University of Sassari, 07100 Sassari, Italy

Temporomandibular joint disorders (TMDs) represent a group of conditions that cause pain and dysfunction in the joints and muscles responsible for jaw movement [1]. These disorders can be an extraordinary burden, not just in terms of individual distress and reduced quality of life, but also for society, due to the incurred healthcare costs and loss of productivity in affected patients [2].

The societal implications of TMDs are substantial and often underestimated. The National Institute of Dental and Craniofacial Research estimates that over 10 million Americans are affected by TMDs [3]. The disruption inflicted by TMDs extends from the individual to the societal level, with a notable economic impact arising due to treatment costs and loss of workdays. Thus, persistently neglecting TMDs has the potential to exacerbate economic inequality and undermine societal progress [4].

On an individual level, TMDs are associated with reduced quality of life. The symptoms—persistent pain in the jaw, headaches, and difficulty eating and speaking—are distressing enough to dampen the spirits of even the most resilient patients [5]. Moreover, TMDs are often correlated with other health issues like chronic fatigue syndrome, fibromyalgia, and sleep disorders, which further compound the physical and emotional suffering experienced by patients [6,7].

In light of these significant challenges, it is paramount to adopt a treatment approach that is effective and cost-efficient and poses minimal risk to patients. While surgical approaches are hindered by certain complications [8,9,10,11], conservative and non-invasive therapies possess all these characteristics, and for this reason, now represent the first line of treatment for TMDs [12,13].

The conservative and non-invasive approach to TMD treatment is generally characterized by its emphasis on risk minimization and patient education. Rather than opting immediately for invasive procedures, the first line of therapy focuses on empowering patients by providing knowledge about their condition and equipping them with tools to effectively manage their symptoms [12].

At the heart of this approach are physical and behavioral therapies. Physical therapy exercises can enhance jaw mobility, strengthen jaw muscles, and alleviate pain, while behavioral therapies are designed to help patients cope with their pain and may reduce stress, which is known to exacerbate TMDs [14,15].

Pharmacological treatments can also play a role in this conservative approach, as long as they are used judiciously. Non-steroidal anti-inflammatory drugs (NSAIDs) or muscle relaxants are often recommended to alleviate pain and muscle tension in the short term. However, these are not generally considered a long-term solution due to their potential side effects, and are best used as part of a broader, multifaceted treatment plan [16,17].

Dental appliances, such as occlusal splints or bite guards, are another key component of the conservative treatment arsenal. These devices can help protect the teeth from grinding or clenching—common habits amongst TMD patients—and can contribute to reducing muscle tension and pain [18,19].

Importantly, conservative treatments underscore the importance of counseling, self-care practices, and lifestyle modifications, which can have a considerable impact on TMD symptom management. Encouraging patients to adopt healthy habits, such as maintaining a soft food diet, practicing good posture, and avoiding extreme jaw movements, can play a critical role in managing and possibly preventing the progression of TMDs [20,21,22]. Overall, the conservative and non-invasive treatment strategy’s significance lies in its patient-centered approach. By prioritizing patient education, self-management, and non-invasive techniques, this approach respects patients’ autonomy and supports their overall well-being. Therefore, it represents the first line of defense in the battle against TMDs, emphasizing prevention, comprehensive care, and the fostering of a patient-empowered path to recovery.

This Special Issue presents a comprehensive summary of all these research areas. De Almeida et al. [23] compared the effectiveness of arthrocentesis followed by hyaluronic acid infiltration with that of mandibular exercise therapy in patients with symptomatic disc displacement without reduction; they found that while both treatments had similar impacts on pain and mandibular motion, arthrocentesis followed by hyaluronic acid infiltration demonstrated more significant long-term improvements in the patients’ quality of life. The study by Vaira et al. [24] assessed the quality of TMD information available on YouTube and found it to be generally poor, suggesting that healthcare professionals and academic institutions should correct misinformation and produce high-quality content to guide patients through accurate diagnostic and therapeutic processes. The study by Di Giacomo et al. [25] revealed that dynamic splint therapy was more effective than stabilization splint therapy in increasing maximum jaw opening, reducing pain, and improving functional movements in patients with acute anterior disc displacement without reduction; this is likely due to its greater orthopedic action and joint mobilization. Derwich and Pawlowska [26] found that long-term occlusal splint therapy combined with physiotherapy significantly lowered the position of the hyoid bone and decreased the dimension of the lower part of the oropharynx in patients diagnosed with temporomandibular disorders. In their study, Sikora et al. [27] demonstrated that repeated intra-articular injections of platelet-rich plasma (PRP) into temporomandibular joint (TMJ) cavities effectively reduced joint pain and increased mandibular mobility in patients with TMJ disorders, suggesting that PRP injections are a viable, minimally invasive treatment option for such conditions. The study by Schmitd et al. [28] aimed to identify and discuss controversial topics in guideline development for the early diagnosis and treatment of chronic rheumatoid arthritis of the temporomandibular joint (TMJ); they emphasized the importance of contrast-enhanced MRI for diagnosis and limiting intra-articular corticosteroid injections to therapy-refractory cases, and stated that alloplastic joint replacement is preferable in adults, while autologous reconstruction might be a viable alternative in children/adolescents. The study published by Pihut et al. [29], revealed that the use of ultrasound examinations for temporomandibular joint disorders can provide detailed insights into joint pathologies, thereby influencing diagnoses and treatment planning, with the myofascial pain group showing more pathologies than expected, necessitating additional treatment procedures. Dowgierd et al. [30] proposed a protocol that utilizes 3D virtual surgical planning and custom biomaterials for treating temporomandibular ankylosis; it showed significant improvements in mouth opening and mandible function, favoring gap arthroplasty and aggressive rehabilitation prior to prosthesis placement over costochondral autografts, and was particularly beneficial in pediatric and adolescent patients.

This Special Issue also includes four literature reviews. The review by Pihut and Kulesa-Mrowiecka [31] highlighted the medical emergencies linked to temporomandibular disorders, such as disc displacement without reduction, sudden contraction of the lateral pterygoid muscle, secondary trigeminal neuralgia, myofascial pain syndrome, and joint issues, emphasizing that incorrect treatment can cause permanent damage to the joints’ soft tissue structures. Chęciński et al. [32] reviewed emerging substances for intra-articular injections in the temporomandibular joint to relieve pain and increase mandibular abduction, highlighting the potential promise of bone marrow and adipose tissue injections. González-Sánchez et al. [33] analyzed and compared various physiotherapy treatment techniques for temporomandibular disorders, concluding that a combination of therapeutic exercise protocols and manual therapy techniques typically provides the best results. Finally, Raccampo et al. [34] presented three case studies and a literature review of the rare Jacob’s disease, characterized by pseudojoint formation between an abnormal mandibular coronoid process and the zygomatic bone’s inner surface, and proposed a new classification due to the disease’s varied presentations.

With its broad array of high-quality research and multidisciplinary perspectives, this Special Issue on the conservative treatment of temporomandibular disorders represents a critical and enlightening contribution to this important research field.
